# Using a new interrater reliability method to test the modified Oulu Patient Classification instrument in home health care: Common mistakes and methodological issue

**DOI:** 10.1002/nop2.359

**Published:** 2019-08-08

**Authors:** Mohammad Aghajani, Fatemeh Sadat Asgarian

**Affiliations:** ^1^ Infectious Diseases Research Center Kashan University of Medical Sciences Kashan Iran; ^2^ Student Research Committee Kashan University of Medical Sciences Kashan Iran; ^3^ Trauma Research Center Kashan University of Medical Sciences Kashan Iran

I read the study conducted by Jill Flo and colleagues published in the January 2018 issue of nursing open (Flo, Landmark, Hatlevik, & Fagerström, 2018). The authors tried to evaluate to test the interrater reliability of the modified Oulu Patient Classification instrument, using a multiple parallel classification method based on oral case presentations in home health care in Norway (Flo et al., 2018). They used Kappa coefficient in their analysis (Flo et al., 2018), which is one of the common mistakes in reliability or reproducibility analysis repeatability, or precision is being assessed by different statistical tests such as Kappa coefficients, which is one of the common mistakes in reliability analysis. Two major weaknesses of the kappa value to assess the agreement of a qualitative variable are as follows: it depends on the prevalence of each stratum, which means it can be possible to have a different kappa value having the same percentage for both concordant and discordant cells, and also depends on the number of stratums, which means the higher the number of stratums, the more lesser the kappa value is (Lawrence & Lin, 1989; Rothman, Greenland, & Lash, 2010). Generally, for quantitative variable, intraclass correlation coefficient and, for qualitative variables, weighted kappa should be used with caution because simple kappa has its own limitation (Lawrence & Lin, 1989; Szklo & Nieto, 2007). In conclusion, for reproducibility or precision analysis, appropriate tests should be applied by researchers.

## CONFLICT OF INTEREST

The author reports no conflicts of interest.
